# Improving nutritional status and health outcomes in school-going adolescents: a randomized controlled trial of nutrition and WASH education interventions in Gojra, Pakistan

**DOI:** 10.3389/fpubh.2025.1440634

**Published:** 2025-06-18

**Authors:** Shagufta Perveen, Rozina Karmaliani, Rozina Farhad Mistry, Rubina Barolia

**Affiliations:** School of Nursing and Midwifery, Aga Khan University, Karachi, Pakistan

**Keywords:** adolescents, biomarkers, body mass index, clinical assessment, nutrition, schools

## Abstract

The presence of both undernutrition and over-nutrition within a population is termed the double burden of malnutrition. Among school-going adolescents, prevalent micronutrient deficiencies are iron, iodine, vitamin A, and zinc. This study examined the effect of a school-based nutrition and WASH (Water, Sanitation and Hygiene) education on low body mass index (BMI;<18.5 kg/m^2^) and malnutrition symptoms among adolescents in Gojra. A randomized controlled trial was conducted involving 603 participants from grades 6 to 8, with 321 in the intervention group and 282 in the control group. Pre- and post-intervention assessments included validated questionnaire. Blood tests, anthropometric measurements, BMI evaluations and clinical assessments were performed to identify biochemical markers and cases of malnutrition. Following the intervention, a slight decrease in average BMI was observed (from 16.7 to 16.2 in boys and 18.5 to 17.6 in girls). Stunting increased in the control group but remained stable among intervention group of boys and showed only a slight rise in girls. Clinical improvement was noted in indicators such as hair, nails, eyes, and teeth, although biomarkers remained largely unchanged. In conclusion, the school-based intervention had a positive effect on clinical nutritional indicators and helped prevent further deterioration of nutritional status. While BMI and biomarkers showed minimal changes, the visible health improvements highlight the potential of integrated school health program to address the dual burden of malnutrition in adolescents.

## 1 Introduction

Nutrition is fundamental to physical growth, cognitive development, emotional wellbeing, and diseases prevention across the life course. Life course theory emphasize that early life interventions particularly during as adolescence can produce long term benefits in health, productivity, and diseases prevention (1). A balanced diet During childhood and adolescence supports immunity, maturation, and optimal development (2–5). Adolescence (age 10–19) the second most critical growth phase after early childhood, is marked by increased nutritional demands due to rapid physical and cognitive changes (6).

From a socio ecological perspective, adolescents health behaviors are influenced by individuals knowledge, family and peer dynamics, school environment, and broader community norms (7, 8). Schools are ideal setting for integrated health promotion, where education, peer influence, and family engagement converge. Incorporating nutrition and WASH (Water, Sanitation, and Hygiene) education in school can address multiple determinants of adolescent’s health. Despite this, health programs in many low- and middle-income countries (LMICs), like Pakistan, have historically focused on children under five and maternal health. Early adolescents (ages 10–14) remain underserved, despite their unique vulnerabilities and untapped potential for positive change. The 2018 Pakistan National Nutritional Survey reported high rates of underweight among boys and rising overweight / obesity among girls, reflecting the dual burden of malnutrition. These outcomes are stem not only from poor diets but also from inadequate sanitation, waterborne diseases and limited access to health information and hygiene facilities (9, 10).

Malnutrition in adolescents result from a complex interplay of biological, behavioral, and environment factors. Poor diet quality, micronutrient deficiencies, inadequate hygiene practices, and limited WASH infrastructure contribute to a vicious cycle of infection and undernutrition (11). These issues are compounded by unsafe water, lack of sanitation, and minimal health education (12, 13). Diarrheal diseases, a leading cause of school absenteeism 34% of girls and 40% boys aged 10–14 in Pakistan (14, 15). Evidence suggests that handwashing with soap can reduce diarrheal risk by over 40%, highlighting the importance of school bases hygiene interventions (16).

Micronutrient deficiencies particularly in iron, iodine, vitamin A, and zinc are common South Asia adolescents (17). While some school-based interventions exist, few combine micronutrient supplementations with integrated nutrition and WASH education using evidence based behavioral change strategies. Whole school approaches integrating curriculum, values, infrastructure, and community engagement have shown positive effects on adolescents health outcomes, including reduced anemia, improved growth, and enhanced hygiene practices (8, 18, 19).

In Pakistan, adolescent focused interventions remain scarce, with most programs targeting children under five and pregnant or lactating women. Early adolescents, who make up approximately 12% of the national population (20), are notable underserved. There is a critical gap in research examining the combined effect of micronutrient supplementation and integrated nutrition and WASH education in school settings for this age group. This study evaluates the effect of a school-based nutrition and WASH education intervention, combined with micronutrient supplementation, on the nutritional status of adolescents in grade 6–8 in Gojra, Punjab, Pakistan. We hypothesize that adolescents exposed to the integrated intervention will show significant improvements in nutritional status and hygiene practices compared to their peers in the control group. Aligned with socio-ecological model and Sustainable Development Goals (SDGs 3, 4, 5, and 6) this study is among the first in Pakistan to target early adolescents through an integrated school-based approach. It offers timely evidence to inform policy and future adolescent health strategies.

### 1.1 Objectives of the study

1. To determine the effect of a school-based nutritional and WASH education and micronutrient supplementation intervention to improve the nutritional status among 6–8 grade school-going adolescents.
2. To measure the nutritional outcome of a school-based nutritional, micro-supplementation and WASH education intervention on micro-nutrient levels among 6–8 grade school-going adolescents

## 2 Materials and methods

### 2.1 Study design and sampling

**Figure 1:**
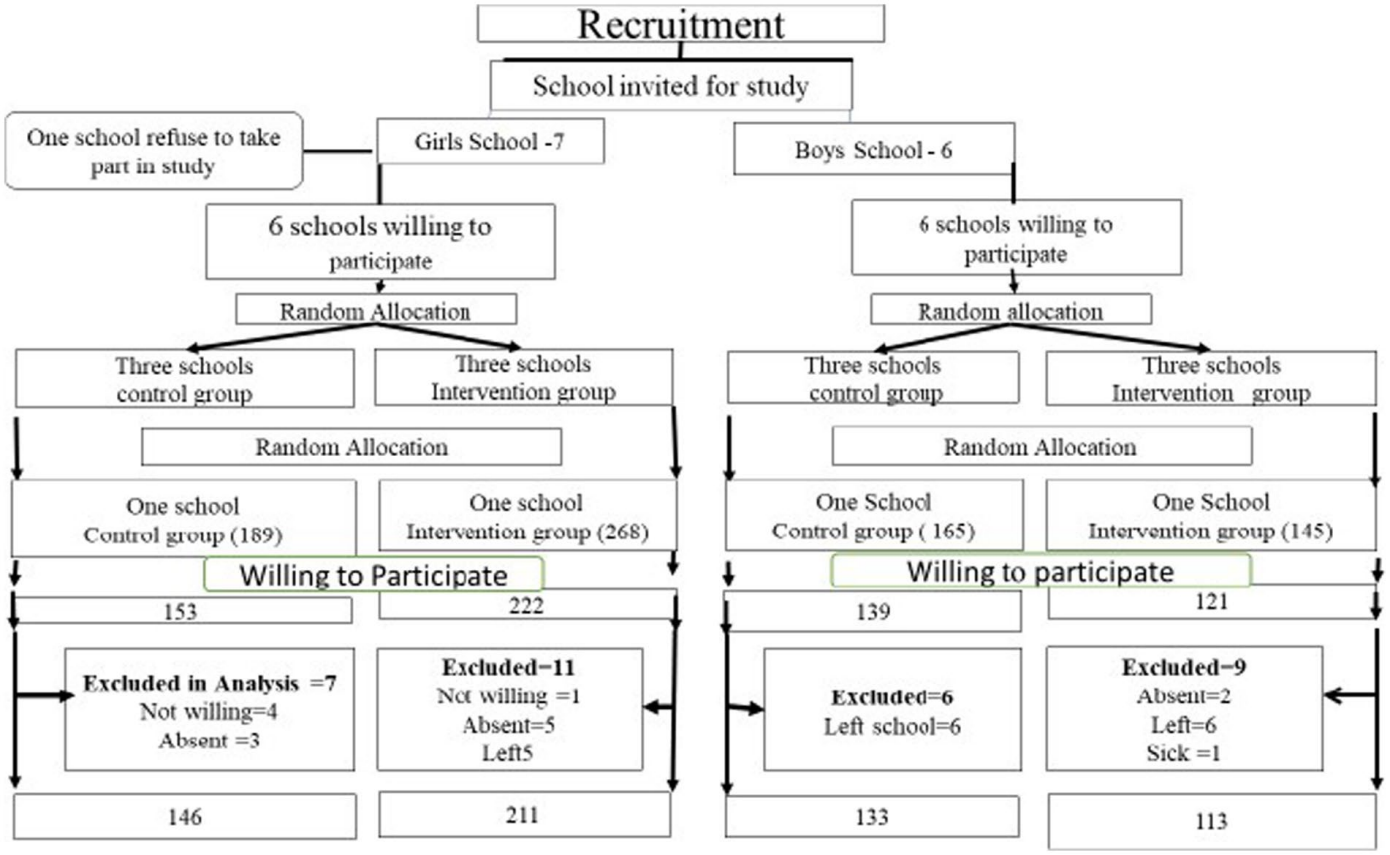
CONSORT diagram.

A randomized controlled trial was conducted to assess the impact of nutrition and WASH education, combined with micronutrient supplementation, on reducing malnutrition among school-going adolescents. The study took place in Gojra, a city located in the Punjab province of Pakistan. The source population consisted of all adolescents enrolled in grades 6, 7, and 8 in public schools within Gojra city, aged 10 to18 years. The sample size was calculated using the Open-Epi formula based on a 42% prevalence of undernutrition among adolescents and a 95% confidence level, which required a sample of 636 adolescents. All public elementary schools for boys and girls in the city were invited to participate. Among these, there were seven boys and six girls’ schools. From the schools willing to participate, two boys’ schools (totaling 260 boys) and two girls’ schools (totaling 375 girls) were randomly selected to achieve the calculated sample size. These four schools (two boys and two girls schools) were then randomized, with one school from each gender category assigned to the intervention group and the other to the control group (see Figure 1). Inclusion criteria includes all adolescent’s aged 10 to 18 years, both boys and girls enrolled in grade 6,7, and 8 grade in public school within Gojra city and were willing to participate in the study, with written informed consent obtained from both the participants and their guardians. All adolescents who were absent from school due to illness or other reasons during the study period were excluded from study.

### 2.2 Instrument

Data were collected using a structured questionnaire adapted for adolescents in grades 6to 8 from schools in Gojra, administered both pre-and post-intervention. The tool compromised two main sections:
*Part A*: Sociodemographic Characteristics –captured age, gender, socioeconomic status, and other relevant demographics.
*Part B*: Nutritional Assessment – informed by literature and qualitative analysis, this section evaluated:

- *Physical Examination*: Assessment of hair, nails, eyes, mouth, and presence of goiter.
- *Body Mass Index (BMI):* calculated from height and weight measurements, adjusted for age and sex using CDC criteria.
- *Biochemical Markers*: included CBC, zinc and calcium level to assess nutritional status.

### 2.3 Data collection

The questionnaire was translated into Urdu and administered individually in classroom to collect participant data. Demographics and anthropometric, clinical, and biomarker data were collected at baseline (September 2021), and were reassessed post-intervention (February 2022). Demographics were recorded only at baseline. Public schools were selected for feasibility based on resource availability, accessibility, and administrative support. Anthropometric measurements (height and weight) were obtained using standard protocols. Height indicator tape ruler was fixed on a wall and adolescents were measured standing straight, without shoes, heels together, and looking straight ahead against the fixed measuring tape. Height was measured to the nearest 0.1 cm. For weight, an analog weighing machine with an adjustable zero was used. Each adolescent was weighed while wearing light clothing and without shoes. Measurements were taken in duplicate, with a third taken if discrepancies arose; the average was used to calculate BMI. Nutritional status was classified using CDC age and sex specific BMI percentiles. Clinical assessment of skin, hair, nails, eyes, oral cavity (teeth and gums), and goiter were conducted using a structured checklist. All assessments were performed by the researcher, a trained nurse with 20 years of clinical experience. Biochemical markers (CBC, serum calcium, and zinc) were analyzed in 20% of the sample (*n* = 27 per group). Blood samples were collected at Aga Khan laboratory with parental consent at both time points to assess nutritional and health status.

### 2.4 Intervention

For the intervention group, a 16-week program was implemented, focusing on nutrition and WASH (Water, Sanitation, and Hygiene) education alongside micronutrient supplementation. The program consisted of 12 weekly nutrition education sessions and 4 WASH education sessions, each lasting 45–60 min.

#### 2.4.1 Nutrition and WASH education

The core component of the nutrition education was based on the Pakistani food pyramid and covered key topics such as the definition and importance of balanced diet, the function of nutrients, the effects of nutrient deficiencies, and methods to obtain essential nutrients while maintain good hygiene. The education sessions involved a combination of classroom activities, interactive discussions, and the distribution of information brochures. The nutrition education sessions were delivered through various engaging methods, including role plays, flip charts, peer presentations, and healthy competitions among adolescents via games. Posters on the food pyramid, personal hygiene, handwashing practices, and the importance of nutrients were displayed in class rooms. The Sessions were facilitated by the researcher in both boys’ and girls’ schools, with the assistance of the respective class teachers. Handwashing demonstrations were conducted in each intervention class, followed by re-demonstrations to reinforce proper techniques. Sessions also included instructions on maintaining personal hygiene.

#### 2.4.2 Micronutrient supplementation

Micronutrient supplementation specifically Iron folate (IFA) tablets, was administered to underweight adolescents (<5th percentile) and those with low Hemoglobin levels. The dosage was 1 tablet per week for 16 weeks and I tablet daily for menstruating girls. The IFA tablets were provided by the district health department and distributed through lady health workers to eligible adolescents at their homes. Regular follow-up calls were made to the mothers or immediate caretakers of the adolescents to ensure weekly IFA tablet intake. No significant side effects were reported from the administration of tablet; however, two participants experienced nausea and vomiting and were referred to local health department services. Additionally, nutrition-related messages tailored to underweight adolescents were shared with mothers or immediate caretakers through the adolescents.

### 2.5 Statistical analysis

Data were analyzed using the Statistical Package for Social Sciences (SPSS) version 23.0. Quantitative data for pre- and post-intervention assessments are presented as means and standard deviations. Comparison between the intervention and control groups, at both pre- and post-intervention stages, were made using paired *t*-tests and chi-square tests. For variables not meeting normal distribution, McNamar’s non-parametric test was used.

### 2.6 Ethical considerations

The study received approval from the Aga Khan University Ethical Review Committee (AKU ERC) (Reference No: 2021–3,572-19378) and ClinicalTrials.gov PRS (Reference No: NCT06009198). Formal approvals were also granted by the Secondary Education Department of Punjab and Chief Executive Officer (CEO) of the health department at the district level. Consent was obtained from the management of the participating schools in Gojra city. For adolescents’ participation, assent was acquired after receiving permission from their parents or immediate caretakers. The trial was conducted in accordance with the approved protocol, Good Clinical Practices (GCP) guidelines, and all applicable regulatory requirements.

## 3 Results

A total of 635 adolescents participated, with a final sample of 603 after excluding 32 withdrawal (Table 1). Participants included 209 boys and 307 girls aged 10–14 years, and 68 girls and 51 boys aged 15–19 years. The mean age was 12.77 ± 1.52 years for girls, and 12.64 ± 1.53 years for boys. Most early adolescents were in grade 6 to 8, while late adolescents were predominantly in grade 8. Family size ranged from 3 to 22, with nearly half (48.7%) having more than four siblings. Parental education varied, with a considerable proportion of fathers and mothers having no formal schooling or only basic literacy.

**Table 1:** Socio-demographics characteristics.

Category		10–14 years		15–19 years	
		Boys (%)	Girls (%)	Boys (%)	Girls (%)
Age		32.9	48.3	8.03	10.07
Class (grade)	6	42.6	8.9	0	11.5
	7	33.8	34.4	23.1	26.9
	8	23.6	36.7	76.9	61.5
Total family members	3–4	3.6	4.1	0	0
	5	16.9	6.8	7.7	3.8
	6	17.4	21.1	23.1	15.4
	≥ 7	60.7	66	69.3	80.8
Siblings of respondent	1	1.5	2.0	0	3.8
	2	5.1	5.1	0	11.5
	3	23.1	10.9	7.7	7.7
	4	21.5	27.6	30.8	23.1
	> 4	48.7	54.4	61.5	53.8
Fathers’ educational status	Illiterate	17.4	15.6	23.1	34.6
	Read and write	12.3	8.5	23.1	19.2
	Grade - 5	17.4	18.0	38.5	3.8
	Grade - 8	16.4	15.0	15.4	7.7
	Grades-10	27.7	30.6	0	26.9
	Grade ≥12	8.7	12.2	0	7.7
Mothers’ educational status	Illiterate	20.0	24.1	30.8	42.3
	Read and write	13.8	9.9	23.1	15.4
	Grade - 5	20.0	19.4	38.5	15.4
	Grade - 8	17.9	15.0	7.7	3.8
	Grades-10	17.9	23.5	0	19.2
	Grade ≥12	10.3	8.2	0	3.8
Fathers’ occupation	Laborer	49.7	42.9	92.3	53.8
	Employed	29.8	21.1	7.7	19.2
	Deceased	3.6	5.1	0	11.5
	Other (not known)	16.9	30.5	0	15.3
Mothers’ occupation	Laborer	7.2	8.5	7.7	15.4
	Employed	2.0	3.1	0	3.8
	Deceased	2.6	1.0	0	0
	House hold	88.1	87.4	92.3	80.7

At the baseline, mean weight, height and BMI were 41.14 ± 9.54 kg, 147.85 ± 7.58 cm, and 18.50 ± 3.63 kg/m2 for girls; and 38.38 ± 8.70 kg, 150.70 ± 10.13 cm, and 16.51 ± 2.74 kg/m^2.^ For boys. Post intervention, BMI decreased slightly in both groups. In the intervention group, boys BMI dropped from 16.7 to 16.2 and girls from 18.5 to 17.6 in the control group, boys BMI reduced from 16.3 to 16.1 and girls from 18.5 to 17.5. A significant difference in BMI changes was observed between groups (*p* < 0.05) (Table 2), with the control group exhibiting a greater reduction, possibility due to confounding factors such as the COVID-19 or environmental changes In early adolescents, the proportion of underweight individuals increased post intervention in both groups. Normal weight prevalence declined among girls, particularly in the control group (39.2 to 29.1%). Overweight and obesity rates decreased across all groups, most notably among intervention girls (8.0 to 1.8%). Among late adolescent, underweight increased in the intervention group (boys: 50 to 66.7%, girls: 42.9 to 52.6%). Normal weight declined in the intervention group but remained unchanged in the control. No obesity was recorded post intervention (Table 3). Stunting increased in early adolescents control participants (boys: 36.0 to 41.0%; girls: 15.0 to 26.7%), while remaining stable in the intervention group (boys: 25.3%; girls: 17.8 to 18.4%). Among late adolescents, stunting rates were unchanged (Table 4).

**Table 2:** BMI index (comparison of pre-and post-assessment findings).

Group	Gender	Pre BMI	Post BMI	*P* Value
Control	All	17.49	16.87	0.00
	Boys	16.3	16.1	-
	Girls	18.5	17.5	-
Intervention	All	17.82	17.09	0.00
	Boys	16.7	16.2	-
	Girls	18.5	17.6	-

**Table 3:** BMI index (as per classification).

Category	Control group				Intervention group			
	Boys (133)		Girls (146)		Boys (113)		Girls (211)	
	Pre (%)	Post (%)	Pre (%)	Post (%)	Pre (%)	Post (%)	Pre (%)	Post (%)
10–14 years								
Underweight	81.0	82.1	54.2	65.8	89.5	91.0	60.3	66.5
Normal	16.0	16.8	39.2	29.1	9.5	9.0	31.6	31.7
Overweight	2.0	1.1	3.3	4.3	1.1	0	8.0	1.8
Obesity	1.0	0	3.3	0.9	0	0	0	0.9
15–19 years								
Underweight	85.7	85.7	0	0	50.0	66.7	42.9	52.6
Normal	14.3	14.3	100	100	33.3	16.7	52.4	47.4
Overweight	0	0	0	0	16.7	16.7	4.8	0
Obesity	0	0	0	0	0	0	0	0

**Table 4:** Stunted: < 3rd percentile.

Category	Control group				Intervention group			
	Boys (133)		Girls (146)		Boys (113)		Girls (211)	
	Pre (%)	Post (%)	Pre (%)	Post (%)	Pre (%)	Post (%)	Pre (%)	Post (%)
10–14 years								
Normal weight	64.0	59.0	85.0	73.3	74.7	74.7	82.2	81.6
Stunted	36.0	41.0	15.0	26.7	25.3	25.3	17.8	18.4
15–19 years								
Normal weight	42.9	42.9	100	100	83.3	83.3	90.5	90.5
Stunted	57.1	57.1	0	0	16.7	16.7	9.5	9.5

Post intervention clinical assessments showed reduced rates of common malnutrition related symptoms among underweight and healthy participants. Underweight boys and girls showed decreased frequencies of diarrhea, hair loss, dry skin, brittle nails, conjunctival pallor, dental issues, fever, body aches, colds. Among healthy participants, boys had fewer symptoms overall, while only diarrhea decreased significantly among girls (Table 5) McNamar’s test indicated significant reductions in malnutrition symptoms such as frequent diarrhea, dry skin, brittle nails, conjunctival pallor, plaque, colds and itching among the intervention group, suggesting positive intervention effects (Table 6).

**Table 5:** Clinical assessment based on underweight and normal weight in all age groups.

Clinical indicators	Underweight				Normal weight			
	Pre		Post		Pre		Post	
	Boys *f*(%)	Girls *f*(%)	Boys *f*(%)	Girls *f*(%)	Boys *f*(%)	Girls *f*(%)	Boys *f*(%)	Girls *f*(%)
Frequent diarrhea	13 (2.0)	22 (3.5)	6 (1.00)	5 (0.83)	3 (0.5)	22 (3.5)	8 (1.33)	17 (2.82)
Hair loss	8 (1.3)	62 (9.8)	0	11 (1.82)	1 (0.2)	62 (9.8)	0	64 (10.61)
Skin and hair appear dry	69 (10.9)	61 (9.6)	15 (2.49)	25 (4.15)	3 (2.0)	59 (9.3)	46 (7.63)	96 (15.92)
Nails appear brittle and break	48 (7.6)	7 (1.1)	9 (1.49)	3 (0.50)	8 (1.3)	3 (0.5)	17 (2.82)	4 (0.66)
Conjunctival Pallor	139 (21.9)	96 (15.1)	31 (5.14)	20 (3.32)	21 (3.3)	59 (9.3)	32 (5.31)	71 (11.77)
Tooth cavities	50 (7.9)	39 (6.1)	20 (3.32)	16 (2.65)	8 (1.3)	33 (5.2)	29 (4.81)	65 (10.78)
Plaque on teeth	154 (24.3)	156 (24.6)	57 (9.45)	42 (6.97)	26 (4.1)	124 (19.5)	102 (16.92)	167 (27.69)
Fever	48 (7.6)	37 (5.8)	17 (2.82)	13 (2.16)	9 (1.4)	32 (5.0)	36 (5.97)	62 (10.28)
Body ache	56 (8.8)	46 (7.2)	11 (1.82)	9 (1.49)	10 (1.6)	36 (5.7)	28 (4.64)	51 (8.46)
Common cold	52 (8.2)	90 (14.2)	43 (7.13)	31 (5.14)	7 (1.1)	68 (10.7)	73 (12.11)	105 (17.41)
Itching	40 (6.3)	27 (4.3)	5 (0.83)	7 (1.16)	7 (1.1)	20 (3.1)	10 (1.66)	22 (3.65)

**Table 6:** Clinical assessment of malnutrition.

Clinical indicators	Control			Intervention		
	Pre %	Post %	McNamar’s test (*p*-value)	Pre %	Post %	McNemar’s test (*p*-value)
Frequent diarrhea	7.8	7.4	1.00	11.8	5.0	0.00
Hair loss	18.7	5.3	0.00	23.3	18.9	0.09
Dry skin	30.4	18.7	0.00	32.6	40.4	0.03
Brittle nails	13.8	7.4	0.01	8.4	4.0	0.01
Conjunctival pallor	50.2	34.3	0.06	49.4	18.3	0.00
Tooth cavities	22.6	28.6	0.16	18.3	15.5	0.06
Plaque on teeth	71.0	73.1	0.60	74.2	15.6	0.00
Fever	23.7	25.4	0.66	16.8	18.0	0.72
Body ache	29.7	20.1	0.01	18.0	13.4	0.07
Common cold	21.2	47.3	0.55	46.0	37.3	0.02
Itching	16.3	8.8	0.04	14.0	6.2	0.00

Laboratory results (Table 7) showed mixed trends. Hemoglobin’s < 11 g/dL slightly increased post intervention (from 8.3 to 12.9%). HCT levels < 35.5% remained stable overall but improved among intervention girls. Zinc deficiency rose from 27.5 to 39.8%, particularly among intervention boys. Iron deficiency anemia decreased in girls but increased slightly in boys. Calcium levels remained stable. Thalassemia prevalence remained low. Post intervention, 12.7% of girls and 3.7% of boys had anemia. Iron deficiency anemia decreased in girls (29 to 20.45%) but rose in boys (18.51 to 20.51%). Zinc deficiency increase in both genders. Overall, despite a decrease in BMI across groups, the intervention group showed relatively better nutrition outcomes. Significant improvements in clinical and anthropometric indicators suggest the effectiveness of the nutrition and WASH education and supplementation intervention.

**Table 7:** Biomarkers (Hb, HCT, zinc, anaemia and thalassemia).

Lab test	Pre-test (*n* = 109)						Post- test (*n* = 93)					
	Hb<11 g/dL	HCT<35.5	Zinc cut off <80 mcg/dl(50–150)	CA mg/dl<9	Iron deficiency A anemia	Thalassemia	Hb<11 g/dL	HCT<35.5	Zinc cut off <80 mcg/dl	CA mg/dl<9	Iron deficiency anemia	Thalassemia
	*f*(%)	*f*(%)	*f*(%)	*f*(%)	*f*(%)	*f*(%)	*f*(%)	*f*(%)	*f*(%)	*f*(%)	*f*(%)	*f*(%)
Control group												
Boys	2 (1.8)	2 (1.8)	8 (7.3)	0	5 (4.6)	0	2 (2.3)	4 (4.3)	9 (9.7)	1 (1.0)	5 (5.6)	1 (1.0)
Girls’	3 (2.8)	4 (3.7)	9 (8.3)	0	5 (4.6)	1 (0.9)	4 (4.3)	4 (4.3)	9 (9.7)	0	6 (6.5)	2 (2.3)
Intervention group												
Boys	0	0	9 (8.3)	0	5 (4.6)	0	1 (1.1)	2 (2.3)	14 (15.0)	0	5 (5.6)	0
Girl’s	4 (3.7)	10 (9.17)	4 (3.7)	0	7 (6.4)	1 (0.9)	5 (5.6)	5 (5.6)	5 (5.6)	0	4 (4.3)	0
Total	9 (8.3)	16 (14.7)	30 (27.5)	0	22 (20.2)	2 (1.8)	12 (12.9)	15 (16.2)	37 (39.8)	1 (1.0)	20 (21.5)	3 (3.3)

## 4 Discussion

The study evaluated the effectiveness of an integrated nutritional and WASH education intervention on malnutrition indicators among early and late school going adolescents. The findings revealed a significant improvement in nutritional outcomes, particularly a reduction in underweight prevalence, reinforcing the value of school-based interventions for promoting adolescent health. These findings are consistent with recent global evidences supporting the effectiveness of multi component school-based strategies in addressing malnutrition (16, 19, 21).

The intervention was delivered to a cohort of 635 adolescents from grades 6 to 8 with a gender distribution skewed toward girls (59%). The similar mean ages of boys and girls (12.64 for boys and 12.77 years for girls) support the representativeness of the sample of adolescents. The significant association between parental education levels (predominately below grade 8) and adolescents nutrition status aligns with prior studies in South Asia and Pakistan, where low parental literacy rate has been linked to higher risk of undernutrition and poor dietary (22).

BMI, a key indicator of nutritional status, showed a more pronounced decline in the intervention group especially among girls than in the control group. This suggests a positive shift towards healthier weight profiles, potentially given by improved awareness of dietary choices. However, it is crucial to consider external influences, such as reduced physical activity during COVID-19 lockdowns, which may have confounded BMI trends. Similar results have been noted in recent post pandemic adolescent nutrition studies (23).

The prevalence of overweight and obesity decreased in the intervention group, with no cases of obesity detected pot intervention. The findings mirror those from a recent studies cluster RCT in Nepal, which demonstrated that school-based nutrition programs can significantly reduce overweight prevalence in adolescents (18, 24). The decrease in overweight prevalence further supports the utility of combining education with behavior focused interventions within school environment.

Stunting trends diverged between groups; while the control group experienced an increase in stunting, rates remained relatively stable in the intervention group. Though the short study duration may limit the ability to detect significant height changes, the stabilization of stunting suggests a protective effect of the intervention. Previous longitudinal studies indicate that consistent exposure to school-based nutrition programs over 12 months or longer is required to significantly reduce stunting (18, 25).

Clinical signs of malnutrition such as diarrhea, hair loss, dry skin, and conjunctival pallor improved post intervention. Particularly among underweight participants. These changes are congruent with findings from a recent study in Jordan, which showed that micronutrient education and dietary modifications led to reduced visible signs of malnutrition in adolescents (21). However, the persistence of suggest that hygiene related determinants, such as water quality and handwashing behavior, require deeper integration into such interventions. This reinforces calls from WASH focused research advocating for structural improvement alongside education (26).

Biomarker results presented a mixed picture. While iron deficiency anemia slightly decreased among girls, zinc deficiency increased in both sexes. These findings may be due to low compliance with supplementation, food based intake limitations, or inhibitory dietary practices (e.g., high tea consumption reducing iron absorption). Recent studies in Bangladesh and India report similar micronutrient gaps despite school-based programs, underscoring the complexity of addressing micronutrient deficiencies without dietary diversification or food fortification (27).

Socio economic factors especially parental education and occupation were significant predictors of nutritional education. Adolescents from better educated household exhibited more favorable anthropometric and clinical indicators, echoing findings from national nutrition survey and reinforcing the socio ecological model underpinning this study (7, 10).

A major challenge was poor adherence to IFA supplementation, often due to low perceived need or understanding. Similar compliance issues have been perceived in LMIC setting, highlighting the importance of community and parental engagement in school health program (9) improved compliance strategies, such as supervised intake and culturally sensitive counselling are recommended in future programs.

## 5 Conclusion

This study provides compelling evidence that nutrition and WASH education and micronutrient supplementation can lead to notable improvements in adolescents’ nutritional status and health outcomes. Through the intervention, reductions in overweight, obesity, and stunting were observed, along with improvements in clinical symptoms of malnutrition. Although, study findings indicated reduction in weight, study findings did not identify any significant change. Despite these positive results, challenges such as non-compliance with supplementation and external factors like the COVID-19 pandemic may have impacted the overall effectiveness of the intervention. It is suggested that future studies may be conducted over a longer period and by addressing all the associated factors as well. Future efforts should focus on addressing these challenges, with a particular emphasis on improving adherence to supplementation protocols and expanding the duration of interventions.

### 5.1 Study strength and implications

This study provides strong evidence that integrated school-based nutrition and WASH education program effectively improve adolescents’ health. Strengths include a diverse sample of 635 students, multiple outcome measures (BMI, clinical signs, and biomarkers), and a comprehensive intervention approach. Notable improvements were observed in nutritional status particularly undernutrition and overweight as well as reduction in clinical symptoms. The findings underscore the effectiveness of multi component, school-based interventions and high light the critical roles of parental education, healthcare support, and compliance. These results have clear policy implications, offering scalable solutions to adolescents’ malnutrition in resource limited setting.

### 5.2 Recommendations

To enhance the effectiveness of interventions addressing adolescent malnutrition, the following recommendations are proposed:

1. To address non-compliance with micronutrient supplementation, increased more involvement of healthcare workers is needed. Educational campaigns targeting both adolescents and their families can dispel misconceptions, fostering better adherence, especially to IFA tablets.
2. Longer intervention periods are recommended to address persistent nutrient deficiencies like iron and zinc and achieve more significant impacts on malnutrition indicators such as stunting and BMI.
3. The government should prioritize safe drinking water and sanitation in schools to prevent waterborne diseases. Posting healthcare workers in schools can provide timely health interventions and strengthen school health infrastructure.
4. Incorporating health and nutrition education into school curricula will help promote healthy eating habits, hygiene, and safe water practices, leading to long-term improvements in student health.
5. Ensuring the availability of essential micronutrient supplements in all schools should be a government priority to address deficiencies and improve adolescent nutrition.

### 5.3 Limitations

- One of the major limitations was the low compliance with micronutrient supplementation. This was partly attributed to cultural beliefs and misconceptions about supplementation, particularly regarding family planning. Future interventions must consider these factors and incorporate strategies to increase acceptance and adherence.
- Laboratory tests were only performed on participants who and their mothers were willing to undergo these tests. This self-selection bias may have skewed some of the findings, particularly regarding the biomarker data, as those who participated might not fully represent the broader adolescent population.
- Also due to budget constraints, laboratory tests (biochemical markers) could only be performed on 108 adolescents, which limits the generalizability of some of the findings related to biomarkers such as hemoglobin, zinc, and calcium levels.
- Political and economic factors were not assessed. These variables may have influenced the outcomes of the intervention, potentially affecting participant behavior and overall program success.
- Additionally, the school educator training was not assessed. These elements are essential for the effectiveness of nutrition and WASH education interventions, and their exclusion may limit the study’s ability to comprehensively evaluate the intervention’s impact across different educational contexts.

## Data Availability

The raw data supporting the conclusions of this article will be made available by the authors, without undue reservation.
